# Adipose-derived mesenchymal stem cells for early hip osteoarthritis: mechanistic insights and possible clinical evidence

**DOI:** 10.3389/fsurg.2026.1915942

**Published:** 2026-09-07

**Authors:** Mario Mosconi, Alice Montagna, Maria Rizzo, Michela Saracco, Laura Caliogna, Micaela Berni, Gianluigi Pasta, Federico Alberto Grassi, Eugenio Jannelli

**Affiliations:** 1 Department of Clinical, Surgical, Diagnostic and Pediatric Sciences, University of Pavia, Pavia, Italy; 2 Orthopedics and Traumatology Clinic, IRCCS Policlinico San Matteo Foundation, Pavia, Italy; 3 Department of Public Health, Division of Orthopedic Surgery, University of Napoli Federico II, Napoli, Italy

**Keywords:** acetabular delamination, mesenchymal stem cells, osteoarthritis, hip, regenerative medicine, stromal vascular fraction (SVF)

## Abstract

**Purpose:**

Hip osteoarthritis (OA) is increasingly diagnosed in younger, active patients, often linked to femoroacetabular impingement (FAI) and associated cartilage lesions such as acetabular delamination. Conventional surgical options, including total hip arthroplasty, are suboptimal in this population due to implant longevity concerns and high functional demands. Adipose-derived mesenchymal stem cells (AD-MSCs) have emerged as a promising biologic alternative, offering regenerative, anti-inflammatory, and immunomodulatory effects with advantages over bone marrow–derived MSCs in cell yield, harvesting ease, and safety.

**Methods:**

We conducted a review of the literature on the possible use of mesenchymal as a treatment for hip osteoarthritis

**Results:**

Processed via manipulative mechanical techniques, AD-MSCs can be delivered intra-articularly to target early- stage OA and focal cartilage damage, aiming to preserve joint integrity and delay progression. Clinical studies report improvements in pain, function, and quality of life, with low complication rates, particularly in Tönnis grade 1 hips. Acetabular delamination, an early and potentially reversible chondral lesion, represents a compelling therapeutic target for biologic intervention.

**Conclusion:**

This new possible therapy will be a new minimally invasive option for joint-preservation, in particular for young patients. Limitations include heterogeneity in protocols, lack of standardized dosing, and scarce long-term randomized evidence. Future research should focus on optimized delivery methods, patient selection, and high-quality trials to establish AD-MSCs as a validated component of hip preservation strategies. Clinical trials are needed to confirm their efficacy and long-term benefits.

## 1 Introduction

Hip osteoarthritis (OA) is a progressive and disabling joint disease characterized by hip cartilage damage and chronic pain. After knee OA, hip OA is the second common form of OA, with a penetrance of 8.55% (1). An interesting study conducted in 2023 on the prevalence of hip OA showed a geographical discrepancy, Europe has the highest rate of hip OA at 12.59%, while Africa has the lowest rate (1.20%), although in Asia and North America the incidence is respectively 4.26% and 7.95% (1). OA affects joints and surrounding tissues, causing serious damage to the cartilage joint. This condition cause disability, pain, loss of function and a decrease of quality of life. In the past, the onset considered due to the “wear and tear”. Biomechanics and chronic overload considered the cause of destruction of articular cartilage and the resulting inflammation. Recent studies have shown how OA etiology is more complex that involves not only mechanical factors but also and biologic factors. In addition, some epidemiological studies have associated numerous different risk factors with the disease etiology, like age, occupations causing repetitive trauma, continuous overuse of joint, isolated joint trauma, physical activities/participation in sports, gender, obesity/lower extremity muscle mass, wearing high-heeled shoes, race, osteoporosis, bone and joint disorders, inflammatory diseases, metabolic and endocrine disorders, genetic factors and low serum levels of vitamin D (2).

Initially considered an older people typical disease, in recent years an increase in hip OA has been observed in young people. Interesting retrospective work involving 290,897 OA patients revealed a significant increase in the percentage of younger individuals under the age of 44 (3). The causes that led to early disease onset are not clear; it is hypothesized that some risk factors, including youthful obesity and joint damage, play a significant role.

To assess the severity of patients' osteoarthritis and the most appropriate clinical treatment, radiographic classification systems such as the Tönnis classification are commonly used. This classification divides hip degenerative changes into four progressive grades. Grade 0 indicates the absence of osteoarthritis; grade 1 refers to mild joint space narrowing, mild labrum at the joint margin, and mild sclerosis of the femoral head or acetabulum; grade 2 identifies the presence of small cysts in the femoral head or acetabulum, increasing joint space narrowing, and moderate loss of sphericity of the femoral head; and finally, grade 3 is characterized by the presence of large cysts, severe joint space narrowing or obliteration, severe femoral head deformity, and avascular necrosis (4).

Several therapeutic approaches are available for the treatment of Hip OA. The most common use are the conservative approaches indicated for the early and intermediate stages of the pathology while the chirurgic approaches are normally used in the advanced stages of the disease as a last resort (5).

Conservative treatments provide symptomatic relief but do not halt disease progression. Most common treatment are Non-Steroidal Anti-Inflammatory Drugs (NSAIDs), physical therapy, corticosteroid and hyaluronic acid injections. These approaches relief pain and joint function but not stop the cartilage damage progression or avoid the joint structural lack.

While surgical alternatives, like microfracture, Autologous Matrix-induced Chondrogenesis (AMIC), arthroscopy for femoroacetabular impingement (FAI), or hip arthroplasty have shown varying degrees of success, with inconsistent long-term outcomes. These treatments improve joint function, but they are not indicated for young or active patients because they are more invasive approaches and they can need revision surgery and a limited longevity (6).

Since the two approaches currently available present obvious differences, new strategies are being studied to bridge the present gap (7, 8).

Thanks to basic and clinical research, in recent decades, new approaches are now being evaluated for the treatment of OA that reduce the progression of the disease and postpone the use of surgical treatment. Between these new therapies we can find orthobiologic approaches, such as hyaluronic acid, platelet-rich plasma (PRP), and biologic preparations based on cell strategies include mesenchymal stem cells and stromal vascular fraction (SVF). These therapies focus to preserving joint integrity, can modify the intra-articular environment and providing symptomatic relief.

Today more innovative is cell therapy. SVF is a heterogeneous mix of cell, using in the last decade in regenerative medicine. It presents potential risks, including possible infections caused by administration, comorbidities related to the collection, possible systemic complications or serious immune reactions, and a potential risk of tumorigenesis, with possible neoplastic formations. Currently, studies in the literature seem to indicate that these risks are minimal and there is no correlation between the use of the treatment and serious risks to the patient's health. However, these studies are recent, and further investigations with long-term follow-up are necessary (9, 10).

SVF include different type of cells such as fibroblasts, preadipocytes, monocytes, lymphocytes, macrophages, pericytes related to angiogenesis and mesenchymal stem cells (MSCs) (11). MSCs have an important role in this context because they show immunomodulatory and anti-inflammatory properties recruiting immune cells to injury sites and inhibiting pro-inflammatory pathways. In addition, they have regenerative characteristics promoting tissue repair through the release of anabolic cytokines and direct differentiation into specialized cells.

Adipose-derived mesenchymal stem cells (AD-MSCs) are a type of mesenchymal cells that offer different advantages: high cell yield, low donor-site morbidity, ease of harvest and suitability for autologous use. In addition, they show anti-inflammatory, immunomodulatory, paracrine, and tissue-repair characteristics that can be indicated to avoid cartilage degradation, that other orthobiologic approaches don't show. These characteristics make AD-MSCs particularly attractive for treating early-stage hip OA and cartilage defects. However, literature studies are limited and need more studies to better understand the effect of this approaches on joint preservation.

This narrative review aims to provide a comprehensive overview of the biological properties, possible clinical applications, and emerging evidence supporting the use of AD-MSCs in the management of hip OA. We also compared this approach with other joint-preserving surgical techniques and intra-articular therapies, highlighting the current limitations, regulatory considerations, and future directions in the field of biologic hip preservation.

## 2 Materials and methods

A narrative review of the literature was performed on two medical electronic databases: PubMed and Embase. The authors per-formed the evaluation of the studies and the data extraction. All studies for this review were selected by following these inclusion criteria:

- All studies were written in the English language
- All studies were an available full text
- All studies were published from 2015 to 2025

The study selection and the data extraction were performed independently by authors. All discrepancies (disagreement) were discussed between the authors and the senior investigators revised the work.

## 3 Result

### 3.1 Preclinical and mechanistic evidence supporting the use of AD-MSCs in osteoarthritis

Adipose-derived mesenchymal stem cells (AD-MSCs) are multipotent stromal cells able to differentiate into different lineages, such as chondrocytes, osteoblasts, and adipocytes. AD-MSCs are present in SVF of adipose tissue and show mesenchymal stem cells markers (CD73, CD90 and CD105) and not present hematopoietic markers (CD34 and CD45) (6, 12). The most important feature of these cells is their paracrine activity, because allow to secrete several bioactive molecules that influence surrounding cells. These factors are called “secretome” and includes chemokines, growth factors, cytokines and extracellular vesicles.

AD-MSCs have immunoregulatory and anti-inflammatory properties, interfering with human immune system in the secretion of a different cytokines. They also show immunomodulatory properties because they have low Major Histocompatibility Complex expression levels (13).

In the OA joint, this immunomodulation can mitigate synovitis and delay cartilage degradation. AD-MSCs are widely used as therapeutic treatments for cartilage and bone defects diseases due to their characteristic and ability to interfere with the secretion of cytokines and growth factors involve in damaged tissues regenerative process (14). For their characteristic they are of great interest in tissue engineering and in regenerative medicine. These cells show a high secretory capacity of different factors, such as cytokines and growth factors, involved in tissue regeneration processes. The most highly expressed cytokines are IL-6, which has both pro and anti-inflammatory effects linked to the environment, TNF-α and IL-1β, which interfere with adipogenic differentiation. Furthermore, AD-MSCs are involved in pathways related to neurogenesis, healing and cell survival, and angiogenesis, through the secretion of platelet-derived growth factor (PDGF), transforming growth factor-*β* (TGF-*β*), fibroblast growth factor-2 (FGF-2), and vascular endothelial growth factor (VEGF) (15).

In addition to have an active role in regeneration process, AD-MSCs have a crucial role in local repair response, with secretion of numerous chemokines, such as SDF-1, MCP-1, and IL-8. In AD-MSCs secretome are present also extracellular vesicles, that contain regulatory microRNAs, bioactive proteins, and signaling lipids. These nanosized structures can promote cell proliferation, stimulate angiogenesis, and inhibit inflammation.

In summery AD-MSCs can interfere with different signaling pathways, including the PI3 K/Akt, MAPK/ERK, STAT3, and NF-*κ*B pathways, involve in inflammatory response, cell survival and proliferation, and immunomodulation pathways (16).

### 3.2 Clinical evidence in hip osteoarthritis

In recent years, adipose-derived mesenchymal stem cells have represented an important new development in regenerative medicine for early-stage hip osteoarthritis. The use of these cells could be an innovative treatment that could preserve joint integrity, delaying or even avoiding surgical interventions. This treatment could be indicated for patients with preserved joint space and mild radiographic changes, typically classified as grade 0 or 1 according to the Tönnis scale (17, 18).

Some recent clinical studies have demonstrated that these cells are able to significantly reduce pain and improve joint function. These benefits were assessed using orthopedic scores and quality of life questionnaires, demonstrating significant improvements within six to twelve months of treatment and maintaining efficacy for up to one to two years (19).

**Figure 1:**
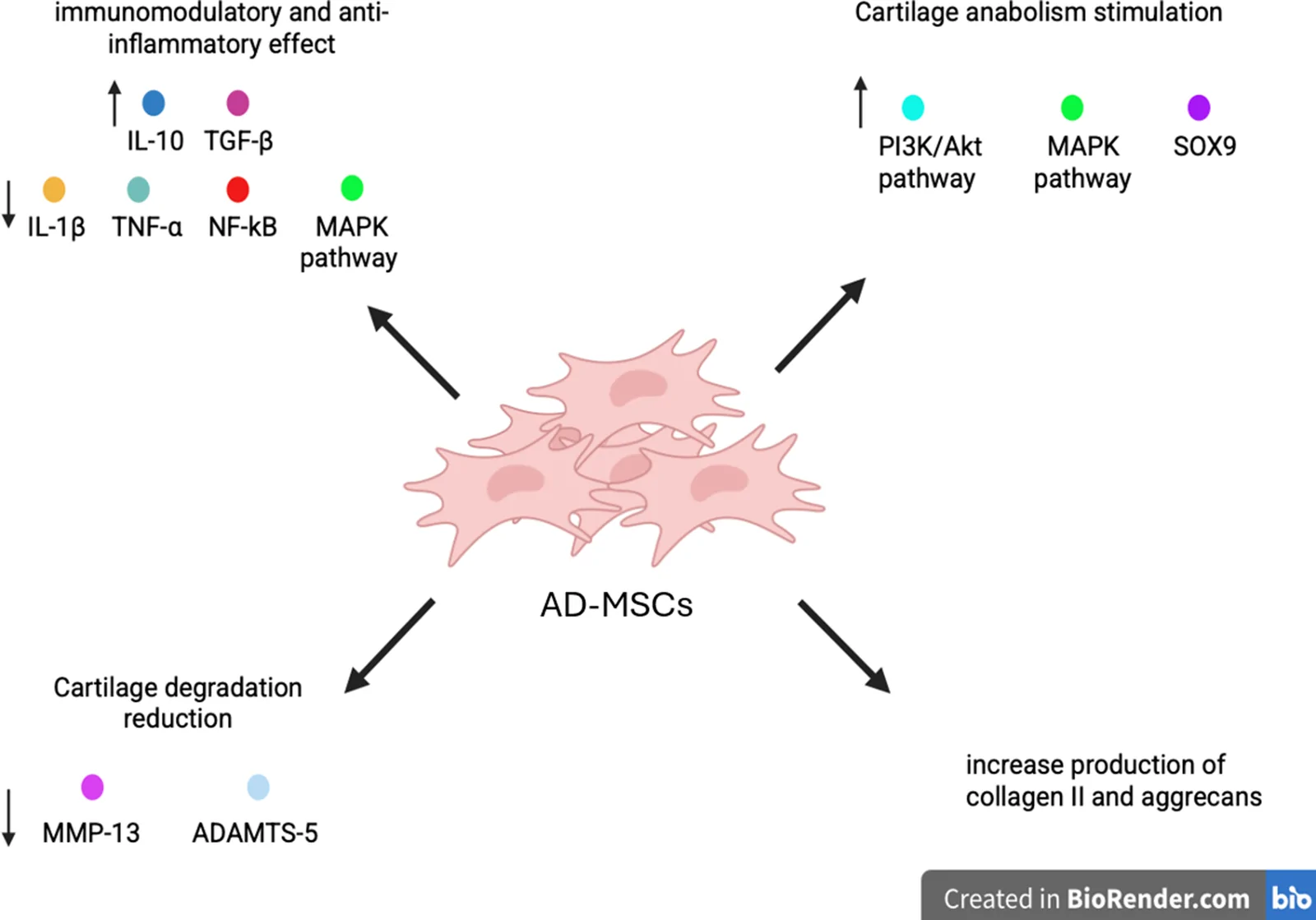
Action mechanism of AD-MSCs in the hip.

In addition to pain relief, AD-MSCs appear to positively impact tissue regeneration, although the mechanisms involved are not entirely clear. AD-MSCs appear to promote macrophage polarization toward the M2 phenotype, which is typically associated with repair. Indeed, they secrete several anti-inflammatory cytokines that inhibit the NF-*κ*B pathway and the expression of pro-inflammatory mediators such as IL-1β, TNF-α, and IL-6, and secrete anti-inflammatory cytokines such as IL-10 and TGF-*β* (13). AD-MSCs also appear to have a positive effect on cartilage repair by modulating the Wnt/*β*-catenin and PI3 K/Akt signaling pathways, which are essential for chondrocyte survival and improve extracellular matrix synthesis (20). They also influence SOX9-mediated signaling pathways (21), promoting the expression of type II collagen and aggrecan, and inhibiting catabolic enzymes such as MMP-13 and ADAMTS-5. AD-MSCs show a close interaction with endothelial cells (ECs), supporting angiogenesis and maturation of ECs and their network, through the secretion of pro-angiogenic cytokines and pericyte-like integrations (13, 21) (Figure 1).

Importantly, AD-MSC therapy is repeatable and may be used in combination with other treatments. Some protocols allow for re-injection after one to two years if symptoms recur, and others integrate biologic therapy into surgical pathways, such as following arthroscopic debridement or osteoplasty, in an effort to enhance cartilage protection and postoperative recovery (22).

Overall, AD-MSCs represent a promising and biologically rational approach for managing early hip OA, with potential benefits in pain control, functional recovery, and molecular modulation of disease pathways. However, longer-term studies and randomized controlled trials are needed to validate these effects and define optimal patient selection (17).

### 3.3 Clinical evidence extrapolated from knee OA or other anatomical sites

Nowadays in literature there are several clinical evidence regarding the benefit of adipose-derived cell-based therapies in knee osteoarthritis, more solid and certain compared their use in hip osteoarthritis. Some studies demonstrate that the use of AD-MSCs can reduce pain, improve function and quality of life, and in some cases promote cartilage regeneration. These benefits derive from the ability of cells to secrete anti-inflammatory factors, modulate the immune response, reduce synovial inflammation, and stimulate tissue and cartilage matrix repair (12). Furthermore, the use of cell therapy appears to be not only effective but also safe, as demonstrated by Freitag et al. in their observational study conducted on 329 patients with a 24-month follow-up. This study did not highlight any adverse effects related to AD-MSC treatment; the only complications observed were limited and manageable with analgesics and ice, likely due to the therapy administration procedure (23).

An interesting study by Fujita et al. demonstrated the positive effects of using stromal vascular fraction (SVF) in knee osteoarthritis, leading to pain reduction, improved range of motion, and decrease cartilage degradation. Furthermore, the study show greater efficacy with two SVF injections instead of one (24).

These results provide promising data on the use of AD-MSCs in the treatment of OA, although data not directly conducted on the hip joint offer clinical support for the potential efficacy of these therapies, providing a safety profile and biological activity. However, these results cannot be directly extended to the hip due to differences in joint anatomy, biomechanics, disease phenotype, and accessibility to treatment. Therefore, specific clinical studies are needed to establish whether the benefits observed in other anatomical regions can be reproduced at the hip joint.

### 3.4 Comparison the use of AD-MSCs versus intra-articular injections

There are several conservative treatments for early hip osteoarthritis, including NSAIDs, physiotherapy, and intra-articular injections of corticosteroids or hyaluronic acid. These treatments tend to provide temporary relief as they do not halt the progression of osteoarthritis but merely alleviate symptoms.

Corticosteroids reduce pain by suppressing pro-inflammatory cytokines (IL-1β, TNF-α) and prostaglandins. This cytokines reduction is mediated by suppressing the NF-*κ*B and AP-1 signaling pathways through activation of glucocorticoid receptors (GRs). Repeated corticosteroid injections can cause chondrocyte apoptosis by downregulating anabolic factors such as SOX9, resulting in matrix loss and accelerated cartilage degeneration (25).

Hyaluronic acid (HA) injections help restore synovial fluid viscosity and reduce mechanical stress. This effect is due to the interaction with the CD44 receptor, present on both synoviocytes and chondrocytes, which modulates the PI3 K/Akt and MAPK molecular pathways. The benefits obtained with these injections are generally observed in the early stages of osteoarthritis but dramatically decline as the disease progresses (6).

Platelet-rich plasma (PRP) is another possible conservative treatment due to its ability to reduce inflammation by inhibiting the NF-*κ*B pathway and activating the PI3 K/Akt and ERK1/2 MAPK pathways. PRP's ability to induce chondrocyte proliferation, angiogenesis, and matrix synthesis is due to the pool of growth factors it contains, including VEGF, IGF-1, TGF-*β*, and PDGF (6). This treatment is indicated for young patients with a Tönnis grade of 0 or 1and presents variable results due to the great heterogeneity resulting from the preparation method (6).

AD-MSC therapy is different from these approaches because it shows a paracrine and immunomodulatory effect due to the stromal cells able to interact with microenvironment.

Comparative studies between AD-MSCs, PRP, hyaluronic acid and other orthobiologic approaches in hip osteoarthritis remain limited so it is difficult to decide which is the best approaches. We can consider AD-MSC a possible promising option, but we need more clinical trial to better understand their role and effect.

Innovative biological approaches appear to be a valid alternative to conservative treatments since, in addition to alleviating symptoms, they appear to influence the progression of the disease.

Therefore, from the data in the literature, biological approaches could be a valid alternative to conservative treatments since, in addition to alleviating symptoms, they appear to influence the progression of the disease (Table 1).

**Table 1:** Current symptomatic, orthobiologic and regenerative approaches for early hip osteoarthritis.

Treatment	Objective	Characteristics	Limitations	Potential advantages of AD-MSCs
NSAIDs	Pain and symptom control	Reducing symptoms	Provides symptomatic but does not avoid disease progression	AD-MSCs may provide not only symptomatic improvement but also anti-inflammatory, immunomodulatory, and tissue-repair effects
Physical therapy	Improvement of pain and joint function	Maintaining function and reducing symptoms	Primarily symptomatic; does not directly address cartilage degeneration	AD-MSCs may additionally modulate the joint microenvironment and support tissue repair
Intra-articular corticosteroids	Reduction of pain and inflammation	Suppression of inflammatory signaling, including NF-*κ*B through glucocorticoid receptor activation	Repeated administration may have potential chondrotoxic effects	AD-MSCs may simultaneously modulate inflammatory, immunomodulatory, and tissue-repair pathways
Hyaluronic acid	Symptom relief and modulation of joint environment	Interaction with CD44 and activation of MAPK/PI3K-related signaling	Clinical efficacy in hip OA remains inconsistent	AD-MSCs provide broader paracrine, immunomodulatory, and tissue-repair activity
Platelet-rich plasma (PRP)	Modulation of the joint environment and stimulation of anabolic pathways	Provides growth factors including TGF-*β*, PDGF, and VEGF and influences ERK/Akt signaling	Considerable heterogeneity in preparation and clinical protocols	AD-MSCs may act simultaneously through inflammatory, anabolic, anti-apoptotic, and immunomodulatory mechanisms
AD-MSCs	Joint preservation, symptom improvement, and potential tissue repair	Paracrine, anti-inflammatory, immunomodulatory, and tissue-repair effects; modulation of NF-κB, PI3 K/Akt, MAPK/ERK, and SOX9-related pathways	Limited clinical evidence, heterogeneous protocols, lack of standardized dosing, and scarce long-term randomized evidence	/

### 3.5 AD-MSCs and acetabular delamination

Acetabular cartilage delamination is an often-overlooked form of cartilage damage in the hip, particularly prevalent among patients with femoroacetabular impingement (FAI). This lesion typically occurs in the anterosuperior quadrant of the acetabulum, at the chondrolabral junction, and is characterized by separation of the articular cartilage from the subchondral bone.

Delamination shows a loss of intermediate cartilage, in which the matrix is ​​disrupted but not yet completely eroded (26). 35%–40% of patients undergoing hip arthroscopy exhibit cartilage delamination, especially younger and more active individuals. This condition is frequently associated with other intra-articular pathologies, such as acetabular labral tears and degeneration of the round ligament.

Histologically, delaminated cartilage shows marked hypocellularity, disorganization of the collagen network, and the presence of vertical cracks indicating early matrix failure and structural vulnerability (26).

At the molecular level, these changes are linked to altered signaling within mechanotransduction pathways, including dysregulation of the integrin/FAK-MAPK axis, which triggers catabolic responses to abnormal joint loading. Furthermore, increased activity of matrix-degrading enzymes, such as MMP-13 and ADAMTS-5, induced by IL-1β and TNF-α through the NF-*κ*B and p38 MAPK pathways, accelerates extracellular matrix degradation. Additionally, oxidative stress and mitochondrial dysfunction promote chondrocyte apoptosis through caspase-dependent mechanisms and downregulation of SOX9, further compromising matrix repair capacity (7).

Delamination plays a central role in the pathophysiological cascade leading to osteoarthritis. It compromises cartilage load-bearing capacity, facilitates fluid ingress into subchondral bone, and contributes to progressive joint degeneration if left untreated (7).

At the subchondral level, an altered interaction between the TGF-*β*/SMAD and Wnt/*β*-catenin signaling pathways can trigger bone remodeling, sclerosis, and angiogenesis, creating a microenvironment that further destabilizes cartilage integrity (20).

Given its location and structural characteristics, delamination often escapes detection on standard radiographs and may only be evident with advanced imaging techniques or during arthroscopic examination. These characteristics make hip delamination a pathological condition that could offer a potential new therapeutic solution with AD-MSCs thanks to their paracrine immunomodulatory effects (which enhance anti-inflammatory cytokines such as IL-10 and TGF-*β*) and their extracellular vesicles rich in microRNAs (e.g., miR-140, miR-21), which can directly suppress catabolic gene expression and support cartilage regeneration. AD-MSCs also stimulate anabolic pathways by activating PI3 K/Akt and ERK1/2 MAPK, which promote chondrocyte proliferation, inhibit apoptosis, and restore SOX9-mediated expression of type II collagen and aggrecan (27).

The use of microfragmented adipose tissue in patients with acetabular delamination has shown promising short- and medium-term improvements in pain and function (26). These findings suggest that delamination, as an early stage of matrix impairment with preserved but structurally weakened cartilage, may be particularly responsive to regenerative therapies (28). There is no direct clinical evidence demonstrating that AD-MSC therapy reverses or structurally repairs acetabular delamination. The available evidence is primarily indirect, and further clinical trials are needed.

## 4 Discussion

Early-onset hip osteoarthritis is a pathological condition that significantly impacts healthcare. In recent years, research has focused on identifying potential new treatments that prevent disease progression and delay or avoid joint replacement. The advent of new biological therapies, which use cell therapy to preserve the joint and delay surgical treatment, have shown promising results. The use of AD-MSCs as a potential biological treatment for early-stage OA has shown improvements in symptoms and joint function.

AD-MSCs exhibit immunomodulatory properties and paracrine effects, modulating the NF-*κ*B and p38 MAPK signaling pathways to suppress pro-inflammatory cytokines (IL-1β, TNF-α, IL-6) while simultaneously promoting anti-inflammatory mediators such as IL-10 and TGF-*β*. Furthermore, AD-MSCs positively interact with PI3 K/Akt and ERK1/2 MAPK signaling, stimulating chondrocyte survival and proliferation and the expression of type II collagen and aggrecan, while inhibiting catabolic enzymes such as MMP-13 and ADAMTS-5.

Compared to other intra-articular injections, such as corticosteroids, hyaluronic acid, or platelet-rich plasma, adipose tissue-derived MSCs may offer an innovative therapeutic route with fewer side effects than other currently available pharmacological treatments. Corticosteroids act primarily through glucocorticoid receptor-mediated inhibition of NF-*κ*B but carry the risk of chondrotoxicity with repeated use. Hyaluronic acid, on the other hand, restores viscoelasticity and signals through CD44, activating the MAPK/PI3 K pathways. However, its clinical efficacy in the hip is inconsistent. Finally, PRP delivers growth factors such as TGF-*β*, PDGF, and VEGF, stimulating ERK and Akt signaling, but presents a high level of heterogeneity in its preparation. In contrast, AD-MSCs act simultaneously on inflammatory, anabolic, and anti-apoptotic pathways, with greater potential for long-lasting benefits and disease modifying effects.

In the presence of conditions that alter normal joint anatomy and biomechanics—such as femoroacetabular impingement or hip dysplasia—correcting the deformity is essential and must be performed prior to AD-MSC treatment. This ensures superior clinical outcomes and long-lasting results, while reducing the risk of recurrence due to chondral damage (22).

However, the main studies conducted with AD-MSCs involve anatomical sites other than hip, so the available data are indirect but suggest that this approach may be beneficial and a potential therapy for hip OA. Further targeted clinical trials are needed to demonstrate the benefits of AD-MSCs for hip OA.

In addition to the nature of the infiltration, it would be interesting to investigate the administration method. Currently, intra-articular injection is the most commonly used procedure; it would be interesting to enhance it with imaging-guided delivery (ultrasound, fluoroscopy) to ensure precise drug targeting, thus enhancing beneficial effects and reducing side effects. It would also be interesting to further explore current knowledge regarding exosomes and extracellular vesicles (EVs) secreted by MSCs, as they mimic the characteristics of AD-MSCs, successfully inhibiting inflammation and stimulating chondrocyte survival and proliferation, while drastically reducing the risks associated with using live cells.

Combining different treatments could be an interesting option for the treatment of OA. PRP treatment induces increased stem cell proliferation and differentiation, while hyaluronic acid infiltration stimulates an extracellular environment through CD44 signaling. Combining these two approaches with biocompatible scaffolds would allow for greater regenerative efficacy by stimulating several complementary biochemical pathways.

In conclusion, AD-MSCs represent a possible biologically option for the management of early-stage hip osteoarthritis. By acting on multiple molecular pathways—suppressing NF-*κ*B-mediated inflammation, enhancing PI3 K/Akt-mediated cell survival, and restoring SOX9-driven anabolism—these cells have the potential to reduce symptoms and maybe to modify hip degeneration.
